# Type 2 diabetes-associated genetic variants of *FTO, LEPR, PPARg* , and *TCF7L2* in gestational diabetes in a Brazilian population

**DOI:** 10.1590/2359-3997000000258

**Published:** 2017-03-20

**Authors:** Mauren Isfer Anghebem-Oliveira, Bruna Rodrigues Martins, Dayane Alberton, Edneia Amancio de Souza Ramos, Geraldo Picheth, Fabiane Gomes de Moraes Rego

**Affiliations:** 1 Departamento de Análises Clínicas Universidade Federal do Paraná Curitiba PR Brasil Departamento de Análises Clínicas, Universidade Federal do Paraná (UFPR), Curitiba, PR, Brasil; 2 Escola de Ciências da Vida Pontifícia Universidade Católica do Paraná Curitiba PR Brasil Escola de Ciências da Vida, Pontifícia Universidade Católica do Paraná (PUC-PR), Curitiba, PR, Brasil; 3 Departamento de Patologia Básica Universidade Federal do Paraná Curitiba PR Brasil Departamento de Patologia Básica, Universidade Federal do Paraná (UFPR), Curitiba, PR, Brasil

**Keywords:** Diabetes, gestational diabetes mellitus, polymorphisms, genetic variants, genotype

## Abstract

**Objective:**

Gestational diabetes mellitus (GDM) is a metabolic disorder that shares pathophysiologic features with type 2 diabetes mellitus. The aim of this study was to investigate the association of the polymorphisms fat mass and obesity-associated (FTO) rs1421085, leptin receptor (LEPR) rs1137100, rs1137101, peroxisome proliferator-activated receptor gamma (PPARg) rs1801282, and transcription factor 7-like 2 (TCF7L2) rs7901695 with GDM.

**Subjects and methods:**

252 unrelated Euro-Brazilian pregnant women were classified into two groups according to the 2015 criteria of the American and Brazilian Diabetes Association: healthy pregnant women (n = 125) and pregnant women with GDM (n = 127), matched by age. The polymorphisms were genotyped using fluorescent probes (TaqMan^®^).

**Results:**

All groups were in Hardy-Weinberg equilibrium. The genotype and allele frequencies of the studied polymorphisms did not show significant differences between the groups (P > 0.05). In the healthy and GDM groups, the C allele frequencies (95% CI) of the FTO rs1421085 polymorphism were 36.8% [31–43%] and 35.0% [29–41%]; the G allele frequencies (95% CI) of the LEPR rs1137100 polymorphism were 24.8% [19–30%] and 22.8% [18–28%]; the G allele frequencies (95% CI) of the LEPR rs1137101 polymorphism were 43.6% [37–50%] and 42.9% [37–49%]; the G allele frequencies (95% CI) of the PPARg rs1801282 polymorphism were 7.6% [4–11%] and 8.3% [5–12%]; and the C allele frequencies (95% CI) of the TCF7L2 rs7901695 polymorphism were 33.6% [28–39%] and 39.0% [33–45%], respectively.

**Conclusion:**

The studied polymorphisms were not associated with GDM in a Brazilian population.

## INTRODUCTION

Gestational diabetes mellitus (GDM) is a complex metabolic disorder defined as glucose intolerance diagnosed in the second or third trimester of pregnancy ( 1 ). In Brazil, about 7% of pregnant women exhibit GDM and this prevalence is increasing in parallel with the obesity epidemic ( 2 ).

As for type 2 diabetes mellitus (T2DM), the pathogenesis of GDM is associated with insulin resistance owing to a reduction of beta cell function. In GDM, pancreatic beta cells are unable to produce enough insulin to compensate for the insulin resistance that commonly occurs during pregnancy ( 3 , 4 ). GDM and T2DM have similar pathophysiologic features, suggesting that GDM is also a polygenic disease ( 5 ). Therefore, studies on the etiology of GDM have primarily been based on T2DM-associated genetic variants ( 6 ).

**Figure 1:**
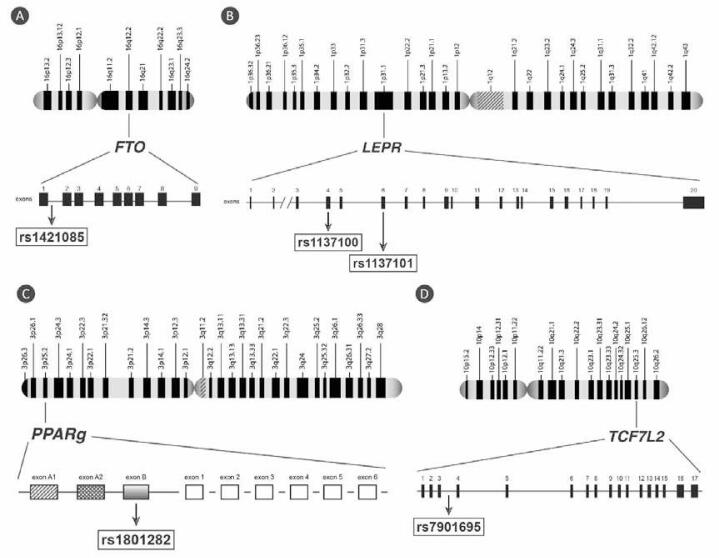
Genomic structure of the studied genes and the location of the selected polymorphisms. rs: reference sequence. (A) FTO: fat mass and obesity associated gene. (B) LEPR: leptin receptor gene. (C) PPARg: peroxisome proliferator-activated receptor gamma gene. (D) TCF7L2: transcription factor 7-like 2 gene.

To further elucidate the genetic mechanisms underlying GDM, we selected several gene polymorphisms previously associated with T2DM: rs1421085 (fat mass and obesity associated; *FTO* ), rs1137100 and rs1137101 (leptin receptor; *LEPR* ), rs1801282 (peroxisome proliferator-activated receptor gamma; *PPARg* ), and rs7901695 (transcription factor 7-like 2; *TCF7L2* ) and investigated their association with GDM. To our knowledge, this is the first study involving these genetic variants and GDM in a Brazilian population. Figure 1 illustrates the genes and polymorphisms studied.

*FTO* is a protein-coding gene located at the chromosome region 16q12.2 and associated with the control of food intake and energy balance ( 7 ). Because of the relationship between *FTO* and obesity, several studies have been conducted to verify the association of *FTO* polymorphisms and T2DM ( 8 , 9 ). In certain populations, *FTO* variants increase susceptibility to T2DM independent of their effect on weight gain, suggesting that changes in the environment or other genetic factors may contribute to the different associations observed between different ethnic groups ( 10 ).

Leptin is a hormone produced by adipocytes and other tissues such as the gastric mucosa, which acts as a signaling molecule in the regulation of body fat mass by negative feedback. Reaching the brain via the bloodstream, leptin acts on hypothalamic receptors thereby reducing appetite, stimulating energy consumption and the loss of body mass as well as the sympathetic nervous system ( 11 ). Leptin exerts its function by binding to leptin receptors (Ob-R). The soluble isoform of the Ob-R receptor, called Ob-Re, is generated by alternative splicing or by proteolytic cleavage of the membrane isoform. Leptin molecules may circulate as the free form or linked to Ob-R. This binding appears to postpone the action of leptin as the leptin-soluble receptor complex increases the half-life of leptin and modulates its action on target cells ( 12 ). Leptin receptors are encoded by the *LEPR* gene ( 13 ).

Leptin receptors are located mainly in the hypothalamus, but are also found in tissues and cells that regulate glucose homeostasis such as pancreatic beta cells. Therein, leptin receptors effect the inhibition of insulin secretion mediated by leptin. This behavior suggests *LEPR* as a candidate gene for association with DM ( 14 ). Consistent with this, *LEPR* was recently identified as a strong candidate for GDM ( 15 ) and was therefore selected for our study.

Peroxisome proliferator activated receptors, known as PPARs, belong to the superfamily of hormonal nuclear receptors that act as transcription factors regulating gene expression. PPARs play a key role in regulating cell differentiation, the metabolism of glucose and lipids, and inflammation ( 16 ). In long periods of hypoglycemia, PPARs are involved in the supply of fatty acids and ketone bodies from adipose tissue as a source of energy. There are 3 subtypes of PPARs encoded by distinct genes: α, β/δ, and γ ( Figure 1 ). PPARγ (PPARg) acts as a mediator of the link between lipid and glucose metabolism ( 17 ). Accordingly, PPARg agonists have been used in the treatment of dyslipidemia and hyperglycemia ( 18 ).

TCF7L2 is a transcription factor involved in stimulating the proliferation of pancreatic beta cells and the production of GLP-1, which stimulates insulin secretion ( 19 ). The *TCF7L2* gene has been associated with T2DM in different populations ( 19 - 21 ). Common variants in intronic region of *TCF7L2* have been identified as strong predictors of T2DM genetic risk ( 22 ). *TCF7L2* polymorphisms modulate blood glucose and insulin secretion and the association of rs7901695 with GDM has previously been described in Caucasians ( 19 ). Another *TCF7L2* polymorphism (rs7903146), which is in linkage disequilibrium with rs7901695, has been associated with GDM in Danish ( 23 ), Australian ( 21 ), Greek ( 5 ), and Swedish ( 20 ) populations.

## SUBJECTS AND METHODS

This cross-sectional study was conducted with unrelated Euro-Brazilian pregnant women who attended a Public Hospital in Southern Brazil. The ethnic group classiﬁcation was based on phenotype (pigmentation of the abdomen, hair color, and type and conformation of the nose and lips). Participants having white skin and European physical characteristics and/or self-reported the European ancestry was classified as Euro-Brazilians ( 24 ).

The Ethics Committee of the Federal University of Parana approved the study and written informed consent was obtained from the subjects.

Euro-Brazilians pregnant women with any gestational period were included in the study. Subjects with kidney (preeclampsia) disease, cardiovascular disease or infection disease were excluded.

The sample (n = 252) was classified into two groups according to the 2015 criteria of the American Diabetes Association ( 1 ) and Brazilian Diabetes Association ( 2 ): healthy pregnant women (Control; n = 125) and patients with GDM (n = 127). The groups were matched by age.

Clinical and anthropometric data were obtained from all subjects. Hypertension was defined according to the VI Brazilian Hypertension Guidelines ( 25 ), which consider hypertension in pregnancy when the systolic blood pressure > 140 mmHg and/or diastolic blood pressure > 90 mmHg. No subjects were under anti-hypertensive treatment. Biochemical parameters were determined by routine laboratory methods using the automated system Architect ci8200 (Abbott Laboratórios do Brasil, Curitiba, PR, Brazil) with reagents, calibrators, and controls as recommended by the manufacturer. 1,5 anhydroglucitol concentrations were measured enzymatically (GlycoMark, Inc., New York, NY, USA). Glycated hemoglobin (HbA1c) was measured using Cobas Integra^®^ 400 Plus (Roche Diagnostica Brasil Ltda., São Paulo, SP, Brazil) with the A1C-2 Tina-Quant^®^ Hemoglobin A1c Gen 2 reagent method certified by the National Glycohemoglobin Standardization Program.

DNA was extracted from whole blood using a modified “salting out” method ( 26 ) and the concentrations were normalized to 20 ng/μL for subsequent assays. Only samples with absorbance ratios (280/260) between 1.8 and 2.0 (NanoDrop, ThermoScientific, Waltham, MA, USA) were used in this study. The polymorphisms were genotyped using real-time polymerase chain reaction (PCR) amplification with fluorescent probes as described in Table 1 . Genotyping experiments were carried out using a 7500 Fast™ Real-Time PCR System (Life Technologies/Applied Biosystems Foster City, CA, USA). The manufacturer provided the reagents (Master Mix^®^ and Genotyping Assay^®^ SNPs) and other real-time PCR materials (Applied Biosystems). The reaction mixture (8 μL final volume) contained 3.0 μL Master Mix (DNA polymerase, Mg2+, buffer, and additives), 0.1 μL SNP Genotyping Assay (40X), 1.9 μL ultrapure water, and 3.0 μL genomic DNA at 20 ng/μL. The cycle sequencing conditions were: 1 cycle of 1 min at 60°C (pre-PCR), 1 cycle of 10 min at 95°C, 45 cycles of 15 s at 95°C followed by 60°C for 90 s, and 1 final cycle of 30 s at 60°C (final extension). The quality of the genotyping was 98% or higher.

**Table 1:** Characteristics of the studied polymorphisms

OMIM number	Gene	Chromosome position	Location	Polymorphism		TaqMan® probe
610966	*FTO*	16q12.2	Intron 1	rs1421085	C>T	C___8917103_10
601007	*LEPR*	1p31.3	Exon 4 Exon 6	rs1137100 rs1137101	A>G A>G	C____518168_20 C___8722581_10
601487	*PPARg*	3p25.2	Exon B	rs1801282	C>G	C___1129864_10
602228	*TCF7L2*	10q25.2– 25.3	Intron 3	rs7901695	C>T	C___384583_10

OMIM: Online Mendelian Inheritance in Man^®^.

References: (46,47).

Normality was tested with the Kolmogorov-Smirnov test. Comparisons of parameters with normal distribution were performed using the Student t-test for independent samples or the Mann-Whitney U test for non-normally distributed variables. Categorical variables were compared using the Fisher exact test (two-tailed) or the Chi-square test, as appropriate. Allele frequencies and Hardy-Weinberg (HW) equilibrium were evaluated by the Chi-square test (http://ihg.gsf.de/cgi-bin/hw/hwa1.pl).

Data analysis was performed using Statistica for Windows 8.0 software (StatSoft Inc., Tulsa, OK, USA), and a probability less than 5% (P < 0.05) was considered significant for all analyses.

## RESULTS

Anthropometric and laboratorial data are presented in Table 2 . The GDM group presented a higher body mass index (32.7 ± 5.0 vs 26.9 ± 6.3 kg/m^2^; P < 0.001). The mean of systolic and diastolic blood pressure (mmHg) for control and GDM groups were 108.8 ± 13.3/68.7 ± 9.2 vs 118.4 ± 12.9/73.8 ± 10.5 (P < 0.001). The frequency of hypertension in subjects with GDM differed from the control group, being higher (14.9% *vs* 4.8%, respectively, P = 0.007). GDM group also had a higher family history of DM (approximately 70%) when compared with Euro-descendant healthy pregnant women from Portugal (16%) ( 27 ) and France (19%) ( 28 ). The median (control *vs* GDM) of fasting glucose and 2-h, 75g glucose, 84 (79–88) vs 88 (81–95) mg/dL and 86.2 (79–102) *vs* 161.0 (149–176) mg/dL, showed higher concentration in GDM group (P < 0.001). In GDM group no one of the patients were under insulin therapy. The lipid profile showed no difference between the groups (P > 0.05), but triglycerides was higher in GDM group, 124.0 (96–171) vs 221.0 (175–270) mg/dL (P < 0.001) ( Table 2 ). The other parameters were within reference range for both groups.

**Table 2:** Anthropometric and laboratory characteristics of the studied groups

Characteristics	Control (n = 125)	GDM (n = 127)	P
Age, years	30.6 ± 4.7	31.9 ± 6.4	0.070
Body mass index^a^, kg/m^2^	26.9 ± 5.0	32.7 ± 6.3	< 0.001
Hypertension, %	4.8	14.9	0.007*
Family history for diabetes, %	–	70.1	–
Fasting glucose, mg/dL	84.0 (79–88)	88 (81–95)	< 0.001**
2-h 75g glucose, mg/dL	86.2 (79–102)	161.0 (149–176)	< 0.001**
HbA1c, %	–	5.6 (5.3–5.9)	–
1,5 anhydroglucitol, µg/mL	11.7 ± 6.9	9.8 ± 5.1	0.060
Total cholesterol, mg/dL	213.9 ± 50.0	224.7 ± 45.6	0.074
HDL-cholesterol, mg/dL	55.4 ± 15.8	56.8 ± 12.5	0.451
LDL-cholesterol, mg/dL	130.9 ± 41.5	123.5 ± 38.9	0.146
Triglycerides, mg/dL	124.0 (96–171)	221.0 (175–270)	< 0.001**
Total protein, g/dL	6.9 ± 0.8	6.4 ± 0.5	< 0.001
Albumin, g/dL	4.2 ± 0.6	3.4 ± 0.4	< 0.001
Creatinine, mg/dL	0.80 (0.7–0.9)	0.70 (0.60–0.72)	< 0.001**
Urea, mg/dL	20.4 ± 5.3	16.1 ± 4.8	< 0.001
Uric acid, mg/dL	3.6 (3.0–3.9)	4.3 (3.7–4.9)	< 0.001**

Values are presented as mean ± SD, median (interquartile range) or %; “ –”: no information available. Control: healthy pregnant women; GDM: gestational diabetes. ^a^ Pregnant BMI calculated at blood collection time. P-values, Student’s t-test (independent variables), * Chi-square test or ** Mann-Whitney U test.

The genotype and allele frequencies of the studied polymorphisms were not significantly different (P > 0.05) between the groups ( Table 3 ). All genotypes were in Hardy-Weinberg equilibrium for both groups (P > 0.05). One-way ANOVA and correlation analysis (Pearson) were applied to identify association and correlation between laboratory biomarkers described in Table 2 and the studied polymorphisms. Genotypes for all polymorphisms, coded 1 (common homozygous), 2 (heterozygous) and 3 (rare homozygous), showed no significance (P > 0.05) for all analysis.

**Table 3:** Genotype and allele frequencies of the genetic variants studied in the Control and GDM groups

Gene/SNP	Genotypes	Control n = 125	GDM n = 127	P
*FTO* rs1421085 (C>T)	TT	53 (42.4)	52 (40.9)	0.420*
	TC	52 (41.6)	61 (48.0)	
	CC	20 (16.0)	14 (11.1)	
	C-allele [95% CI]	36.8 [31–43]	35.0 [29–41]	0.680
*LEPR* rs1137100 (A>G)	AA	70 (56.0)	73 (57.5)	0.687*
	AG	48 (38.4)	50 (39.4)	
	GG	7 (5.6)	4 (3.1)	
	G-allele [95% CI]	24.8 [19–30]	22.8 [18–28]	0.604
*LEPR* rs1137101 (A>G)	AA	43 (34.4)	38 (29.9)	0.228*
	AG	55 (44.0)	69 (54.3)	
	GG	27 (21.6)	20 (15.8)	
	G-allele [95% CI]	43.6 [37–50]	42.9 [37–49]	0.876
*PPARg* rs1801282 (C>G)	CC	107 (85.6)	108 (85.0)	0.851*
	CG	17 (13.6)	17 (13.4)	
	GG	1 (0.8)	2 (1.6)	
	G-allele [95% CI]	7.6 [4–11]	8.3 [5–12]	0.782
*TCF7L2* rs7901695 (C>T)	TT	52 (41.6)	44 (34.6)	0.413*
	CT	62 (49.6)	67 (52.8)	
	CC	11 (8.8)	16 (12.6)	
	C-allele [95% CI]	33.6 [28–39]	39.0 [33–45]	0.209

Genotypes depicted as number (%). 95% CI: Confidence interval of 95%.

All polymorphisms were in Hardy-Weinberg equilibrium.

P: probability. Chi-square test or * Two tailed Fisher’s Exact test.

## DISCUSSION

Elevated BMI is a strong predictor of GDM and insulin resistance ( 29 ). In the present study, pregnant women with GDM were found to be heavier than healthy pregnant women ( Table 2 ). The rate of hypertension in the GDM group was higher than that reported in the literature, which is 5–10% ( 30 , 31 ). However, GDM group showed good glycemic control as assessed by a HbA1c median value of 5.6%.

Notably, pregnant women with a family history for DM are at increased risk for developing GDM and for giving birth to macrosomic children ( 32 , 33 ). GDM also induces a dyslipidemia state that is consistent with insulin resistance ( 34 ); triglycerides concentrations in particular were higher in the GDM group (P < 0.001). Finally, although total protein, albumin, creatinine, urea and uric acid levels differed between the groups (P < 0.001), they were within the reference range for these parameters and none of the subjects exhibited clinical symptoms of kidney disease or others pathologies. Pregnancy affects essentially all aspects of kidney physiology. Glomerular filtration rate (GFR) increases 50% as compared with nonpregnant levels ( 35 ) with subsequent decrease in serum creatinine, urea, and uric acid values ( 36 ). Also, increases in glomerular filtration rate and minor increases in urinary albumin excretion have been reported early in the course of diabetes ( 37 , 38 ). These events, could explain the lower total proteins, albumin, creatinine and urea levels found in GDM compared to control group. The uric acid concentration in GDM was approximately 0.7 mg/dL higher than in control group (4.3 vs 3.6 mg/dL, p < 0.001) ( Table 2 ). This observations has been described in other studies and associated with insulin resistance and hypertension, which predominates in GDM group ( 39 - 41 ).

### FTO rs1421085 polymorphism

The *FTO* rs9939609 predisposes to childhood, adult ( 42 , 43 ) and pregnancy obesity ( 44 ). The *FTO* rs1421085 polymorphism is associated with obesity in adults and in European and Chinese children ( 45 ) and with obesity and T2DM in African Americans ( 46 ). No information is available regarding its frequency in pregnant women or its association with GDM, underlying why this polymorphism was selected for analysis in the present study. However, the *FTO* rs1421085 polymorphism was not associated with GDM in the current population studied, nor with the parameters analyzed. The effect of sample size might have contributed to this result. Furthermore, the physiological effect of the presence of this intronic variant and the reported increased risk of obesity and DM need to be elucidated.

The frequency of the C allele on healthy pregnant women in this study was approximately two to three-fold higher than the frequency reported in European, Chinese, and Japanese populations, whereas it was approximately 5 times lower than that reported in an African populations (HapMap – YRI). Brazilians are an admixture population. This genetic background could explain the differences of alleles frequencies when compared to others populations, even with populations with more similar ancestors such as Caucasians. Table 4 compares the reported risk allele frequencies in healthy subjects, including the current cohort of pregnant women, from different populations.

**Table 4:** Comparison between the allele frequencies of healthy pregnant women from the current study with those obtained from healthy subjects in other studies

*FTO* rs1421085 polymorphism							
Population	Characteristics	n	Genotype (%)			Allele (%)	Reference

			TT	TC	CC	C	
Euro-Brazilian	GDM Controls	127 125	40.9 42.4	48.0 41.6	11.1 16.0	35.0 36.8	Present work
Chinese Han		90	70.7	24.4	4.9	**11.6**	HapMap-HCB
European		180	27.4	53.1	19.5	**16.7**	HapMap-CEU
Japanese		91	67.1	28.2	4.7	**18.8**	HapMap-JPT
Turkish	Obeses Controls	190 97	30.5 28.8	51.1 52.6	18.4 18.6	43.9 **44.8**	(48)
Caucasian	Obeses		29.5	30.5	40	**55.2**	(49)
African		90	86.7	13.3	0	**6.6**	HapMap-YRI

***LEPR* rs1137100 polymorphism**							

**Population**	**Characteristics**	**N**	**Genotype (%)**			**Allele (%)**	**Reference**

			**AA**	**AG**	**GG**	**G**	

Euro-Brazilian	GDM Controls	127 125	57.5 56.0	39.4 38.4	3.1 5.6	22.8 24.8	Present work
African		90	69.6	27.7	2.7	16.5	HapMap-YRI
French	Obeses Controls	877 877	56.7 53.4	37.4 39.2	5.9 7.4	24.6 27.0	(50)
British	Obeses Controls	190 132	54.0 55.0	38.0 39.0	8.0 6.0	27.4 25.8	(51)
European		180	49.6	42.5	8.0	29.2	HapMap-CEU
Brazilians	Hypertension	470	42.8	46.8	10.4	33.8	(52)
Chinese Han		90	2.5	27.5	70.0	83.7	HapMap-HCB
Japanese		91	2.5	40.2	57.3	97.4	HapMap-JPT

***LEPR* rs1137101 polymorphism**							
**Population**	**Characteristics**	**N**	**Genotype (%)**			**Allele (%)**	**Reference**

			**AA**	**AG**	**GG**	**G**	

Euro-Brazilian	GDM Controls	127 125	29.9 34.4	54.3 44.0	15.8 21.6	42.9 **43.6**	Present work
French	Obeses Controls	877 877	31.6 31.6	50.0 47.1	18.4 21.3	43.4 44.9	(50)
British	Obeses Controls	190 132	29.0 28.0	53.0 58.0	18.0 14.0	44.6 42.8	(51)
European		180	25.9	53.6	20.5	47.3	HapMap-CEU
African		90	11.1	58.3	30.6	59.7	HapMap-YRI
Japanese		91	1.2	30.5	68.3	83.5	HapMap-JPT
Chinese Han		90	2.2	17.8	80.0	88.9	HapMap-HCB

***PPARg* rs1801282 polymorphism**							
**Population**	**Characteristics**	**n**	**Genotype (%)**			**Allele (%)**	**Reference**

			**CC**	**CG**	**GG**	**G**	

Euro-Brazilian	GDM Controls	127 125	85 85.6	13.4 13.6	1.6 0.8	8.3 7.6	Present work
French	Mothers with glucose tolerance	1708	80	19	1	10.4	(42)
Danish	GDM Women with glucose tolerance	283 2446	75.8 75.2	22.6 22.7	1.6 2.1	12.8 13.5	(22)
Danish	T2DM Controls	1461 4986	76 75	22 23	2 2	13.0 13.9	(53)
Italian	Peripheral arterial disease Controls	201 201	74.1 84.6	23.9 14.9	2.0 0.5	14.0 8.0	(54)
Scandinavian	GDM Controls	637 1232	73.5 74.5	24.8 24.2	1.7 1.3	14.1 13.4	(19)
Scandinavian Arabs	GDM Controls GDM Controls	400 428 100 122	71.5 74.1 91 86.9	27.7 24.5 9 12.3	0.8 1.4 0 0.8	14.6 13.7 4.5 7.0	(44)
Turkish	GDM Controls	62 100	80.7 84	19.3 16	0 0	19.4 16.0	(41)
Korean	GDM Controls	94 41	94.6 82.9	5.4 17.1	0 0	2.7 8.5	(55)
Greek	GDM Controls	148 107	96.6 93.5	3.4 6.5	0 0	3.0 2.0	(5)
Chinese	GDM Controls	55 173	94.5 90.8	5.5 9.2	0 0	3.0 5.0	(56)
Korean	GDM Controls	869 632	91.7 89.7	8.2 10.0	0.1 0.3	4.0 5.0	(43)

***TCF7L2* rs7901895 polymorphism**							

**Population**	**Characteristics**	**n**	**Genotype (%)**			**Allele (%)**	**Reference**

			**TT**	**TC**	**CC**	**C**	

Euro-Brazilian	GDM Controls	127 125	34.6 41.6	52.8 49.6	12.6 8.8	39.0 33.6	Present work
Chinese Han		90	95.3	4.7	0	2.3	HapMap-HCB
Swedish women	GDM Controls	1102 794	51.2 34.2	34.2 43.1	7.6 11.5	26.5 34.4	(45)
Swedish men	T2DM Controls	825 793	52.6 59.2	39.9 36.0	7.5 4.8	27.5 22.8	(57)
European		180	54.5	34.8	10.7	28.1	HapMap-CEU
Japanese		91	94.2	4.7	0.1	3.5	HapMap-JPT
Caucasian African-American	GDM GDM	- -	- -	- -	- -	30 40	(18)
Spanish	GDM Controls	45 25	38 40	44 52	18 8	40 34	(58)
African		90	27.4	56.5	15.9	44.2	HapMap-YRI

Allele frequencies are presented as % [95% confidence interval]. The frequencies of the minor allele (T) that differ from the confidence interval (95%) for the healthy group of the current study are highlighted in bold.

### LEPR rs1137100 and rs1137101 polymorphisms

The *LEPR* rs1137100 polymorphism (K109R; Lys109Arg; 326A > G) is characterized by an A > G substitution resulting in a change of lysine to arginine at codon 109 ( Figure 1 ). Caucasians with the AA genotype are 2-fold more likely to develop T2DM than those with other genotypes ( 47 ). The *LEPR* rs1137101 polymorphism (Q223R; Gln223Arg; 668A > G) is an A> G substitution resulting in the exchange of glutamine for arginine at position 668 of *LEPR* ( Figure 1 ). The presence of this variant was considered as an independent risk factor for T2DM in Malaysian subjects ( 48 ). Finns with glucose intolerance and the presence of the GG genotype (Finnish Diabetes Prevention Study) showed a higher risk for T2DM compared to those carrying the A allele. The rs1137100 and rs1137101 variants are each located in the region encoding the extracellular domain of the leptin receptor. The exchange of amino acids generated by the variants affects all receptor isoforms and might change the action of leptin toward insulin ( 49 ).

The allele frequency for rs1137100 G reported in the present studied was higher than the rate reported for Africans and significantly lower than that for Asians. The ethnic composition of the sample of this study (Euro-Brazilians) is the probable reason for the similarity with the frequencies reported for the English, French, and Europeans in general ( Table 4 ). Elevated leptin concentrations are associated with adiposity and insulin resistance and it has been reported that individuals with the AA genotype of *LEPR* rs1137100 showed higher concentrations than the G allele carriers. Although the function of this variant has not been elucidated, it was associated with the promotion of a change in the extracellular domain of the receptor that affects the leptin binding affinity ( 50 ). In this study it was not possible to verify the association of the polymorphism with obesity, BMI, or lipid or glucose changes (data not showed).

The frequency of G allele for rs1137101 variant was similar to those reported for Europeans and lower than those of Africans (HapMap). Asian subjects presented a higher frequency of the G allele for rs1137101 than other populations, as shown in Table 4 . The variants rs1137100 and rs1137101 of *LEPR* were not associated with GDM in the studied population, nor with the analyzed parameters described in Table 2 (P > 0.05).

### PPARg rs1801282 polymorphism

The *PPARg* rs1801282 polymorphism results in a substitution of proline for alanine at codon 12 of exon B (C > G; Pro12Ala; Figure 1 ). This polymorphism causes a conformational change in the protein, and the presence of the minor allele is associated with a reduction in the activity of PPARg ( 50 ). The rs1801282 C allele is associated with increased transcriptional activity of PPARg and, consequently, increased sensitivity to insulin. The association with T2DM is controversial, as some studies have found a positive association while others showed that the presence of the variant conferred protection against T2DM ( 51 ). Similarly, some studies found no association of rs1801282 with GDM ( 5 , 23 ). In a study conducted in Turkey, this polymorphism had no effect on the prevalence of GDM or glucose concentrations in pregnant women, but its presence impacted the weight of pregnant women with GDM ( 52 ). The presence of the rs1801282 GG genotype was associated with a higher BMI before pregnancy and a higher pre-pregnancy obesity rate, but was related to a 50% reduction in the risk of developing GDM in a French population ( 53 ).

*PPARg* rs1801282 was not associated with GDM in the current studied population, nor with the analyzed parameters described in Table 2 (P > 0.05). In accordance with the results of our study, no association of the variant with GDM was found in Korean ( 54 ), Scandinavian, or Arab pregnant women ( 55 ). The G allele frequencies observed in this study were similar to the French and greater than those reported for the Arab, Greek, Korean, and Chinese populations, whereas Danish, Scandinavian and Turkish populations showed a higher frequency of the G allele ( Table 4 ). Regional population differences might explain these findings.

### TCF7L2 rs7901695 polymorphism

The T allele of rs7901695 conferred increased risk for GDM in American Caucasians, with an odds ratio of 1.98 ( 19 ). In Swedish patients with GDM the CT and CC genotypes of rs7901695 showed a strong association with GDM even after adjusting for maternal age, number of pregnancies, and family history of DM and HLA-DQ genotypes ( 56 ). Based on this information, we expected to find an association between the rs7901695 polymorphism and GDM in the current studied population, but this was not observed.

The rs7901895 C allele is more common in African populations. The frequency found in healthy pregnant women in the present study is similar to those reported for Europeans in general and for Swedish and Spanish pregnant women. In this study, pregnant women with GDM showed allele frequencies similar to those of Spanish, Caucasian, and African American women with GDM, and slightly higher than those of Swedish women with GDM ( Table 4 ). In contrast, less than 5% of the Asian population carries the C allele. Ethnicity-specific factors might be responsible for these differences.

In conclusion, the *FTO* rs1421085, *LEPR* rs1137100 and rs1137101, *PPARg* rs1801282, and *TCF7L2* rs7901695 polymorphisms were not associated with GDM in a Brazilian population, nor with the other parameters analyzed. The data from this study will likely contribute to the understanding the potential roles of these variants across populations; however, further research is required to identify the underlying factors influencing the risk of GDM in the Brazilian white population.

## References

[1] ADA. (2) Classification and diagnosis of diabetes. Diabetes Care. 2015;38 Suppl:S8-16. 10.2337/dc15-S005 25537714

[2] SBD. Diretrizes SBD. Sociedade Brasileira de Diabetes. 2015. Available from: < http://www.diabetes.org.br/sbdonline/images/docs/DIRETRIZES-SBD-2015-2016.pdf> . Accessed on: Apr. 20, 2016.

[3] Buchanan TA, Xiang AH, Page KA. Gestational diabetes mellitus: risks and management during and after pregnancy. Nat Rev Endocrinol. 2012;8(11):639-49. 10.1038/nrendo.2012.96 PMC4404707 22751341

[4] Baz B, Riveline JP, Gautier JF. ENDOCRINOLOGY OF PREGNANCY: Gestational diabetes mellitus: definition, aetiological and clinical aspects. Eur J Endocrinol. 2016;174(2):R43-51. 10.1530/EJE-15-0378 26431552

[5] Pappa KI, Gazouli M, Economou K, Daskalakis G, Anastasiou E, Anagnou NP, et al. Gestational diabetes mellitus shares polymorphisms of genes associated with insulin resistance and type 2 diabetes in the Greek population. Gynecol Endocrinol. 2011;27(4):267-72. 10.3109/09513590.2010.490609 20540670

[6] Robitaille J, Grant AM. The genetics of gestational diabetes mellitus: evidence for relationship with type 2 diabetes mellitus. Genet Med. 2008;10(4):240-50. 10.1097/GIM.0b013e31816b8710 18414206

[7] Merkestein M, Laber S, McMurray F, Andrew D, Sachse G, Sanderson J, et al. FTO influences adipogenesis by regulating mitotic clonal expansion. Nat Commun. 2015;6:6792. 10.1038/ncomms7792 PMC4410642 25881961

[8] Field SF, Howson JM, Walker NM, Dunger DB, Todd JA. Analysis of the obesity gene FTO in 14,803 type 1 diabetes cases and controls. Diabetologia. 2007;50(10):2218-20. 10.1007/s00125-007-0767-0 PMC2151140 17657473

[9] Legry V, Cottel D, Ferrieres J, Arveiler D, Andrieux N, Bingham A, et al. Effect of an FTO polymorphism on fat mass, obesity, and type 2 diabetes mellitus in the French MONICA Study. Metabolism. 2009;58(7):971-5. 10.1016/j.metabol.2009.02.019 19375760

[10] Larder R, Cheung MK, Tung YC, Yeo GS, Coll AP. Where to go with FTO? Trends Endocrinol Metab. 2011;22(2):53-9. 10.1016/j.tem.2010.11.001 21131211

[11] Oswal A, Yeo G. Leptin and the control of body weight: a review of its diverse central targets, signaling mechanisms, and role in the pathogenesis of obesity. Obesity (Silver Spring). 2010;18(2):221-9. 10.1038/oby.2009.228 19644451

[12] Cammisotto P, Bendayan M. A review on gastric leptin: the exocrine secretion of a gastric hormone. Anat Cell Biol. 2012;45(1):1-16. 10.5115/acb.2012.45.1.1 PMC3328736 22536547

[13] Winick JD, Stoffel M, Friedman JM. Identification of microsatellite markers linked to the human leptin receptor gene on chromosome 1. Genomics. 1996;36(1):221-2. 10.1006/geno.1996.0455 8812446

[14] Emilsson V, Liu YL, Cawthorne MA, Morton NM, Davenport M. Expression of the functional leptin receptor mRNA in pancreatic islets and direct inhibitory action of leptin on insulin secretion. Diabetes. 1997;46(2):313-6. 10.2337/diab.46.2.313 9000710

[15] Zhang Q, He M, Wang J, Liu S, Cheng H, Cheng Y. Predicting of disease genes for gestational diabetes mellitus based on network and functional consistency. Eur J Obstet Gynecol Reprod Biol. 2015;186:91-6. 10.1016/j.ejogrb.2014.12.016 25666344

[16] Varga T, Czimmerer Z, Nagy L. PPARs are a unique set of fatty acid regulated transcription factors controlling both lipid metabolism and inflammation. Biochim Biophys Acta. 2011;1812(8):1007-22. 10.1016/j.bbadis.2011.02.014 PMC3117990 21382489

[17] Janani C, Ranjitha Kumari BD. PPAR gamma gene--a review. Diabetes Metab Syndr. 2015;9(1):46-50. 10.1016/j.dsx.2014.09.015 25450819

[18] Li Y, Qi Y, Huang TH, Yamahara J, Roufogalis BD. Pomegranate flower: a unique traditional antidiabetic medicine with dual PPAR-alpha/-gamma activator properties. Diabetes Obes Metab. 2008;10(1):10-7. 10.1111/j.1463-1326.2007.00708.x 18095947

[19] Stuebe AM, Wise A, Nguyen T, Herring A, North KE, Siega-Riz AM. Maternal genotype and gestational diabetes. Am J Perinatol. 2014;31(1):69-76. 10.1055/s-0033-1334451 PMC3884679 23456907

[20] Shaat N, Lernmark A, Karlsson E, Ivarsson S, Parikh H, Berntorp K, et al. A variant in the transcription factor 7-like 2 (TCF7L2) gene is associated with an increased risk of gestational diabetes mellitus. Diabetologia. 2007;50(5):972-9. 10.1007/s00125-007-0623-2 17342473

[21] Freathy RM, Hayes MG, Urbanek M, Lowe LP, Lee H, Ackerman C, et al. Hyperglycemia and Adverse Pregnancy Outcome (HAPO) study: common genetic variants in GCK and TCF7L2 are associated with fasting and postchallenge glucose levels in pregnancy and with the new consensus definition of gestational diabetes mellitus from the International Association of Diabetes and Pregnancy Study Groups. Diabetes. 2010;59(10):2682-9. 10.2337/db10-0177 PMC3083839 20682688

[22] Cauchi S, El Achhab Y, Choquet H, Dina C, Krempler F, Weitgasser R, et al. TCF7L2 is reproducibly associated with type 2 diabetes in various ethnic groups: a global meta-analysis. J Mol Med (Berl). 2007;85(7):777-82. 10.1007/s00109-007-0203-4 17476472

[23] Lauenborg J, Grarup N, Damm P, Borch-Johnsen K, Jorgensen T, Pedersen O, et al. Common type 2 diabetes risk gene variants associate with gestational diabetes. J Clin Endocrinol Metab. 2009;94(1):145-50. 10.1210/jc.2008-1336 18984664

[24] Krieger H, Morton NE, Mi MP, Azevedo E, Freire-Maia A, Yasuda N. Racial admixture in north-eastern Brazil. Ann Hum Genet. 1965;29(2):113-25. 10.1111/j.1469-1809.1965.tb00507.x 5863835

[25] Sociedade Brasileira de Cardiologia; Sociedade Brasileira de Hipertensão; Sociedade Brasileira de Nefrologia. VI Brazilian Guidelines on Hypertension. Arq Bras Cardiol. 2010;95(1 Suppl):1-51. 21085756

[26] Lahiri DK, Nurnberger JI, Jr. A rapid non-enzymatic method for the preparation of HMW DNA from blood for RFLP studies. Nucleic Acids Res. 1991;19(19):5444. 10.1093/nar/19.19.5444 PMC328920 1681511

[27] Ribeiro AMC, Nogueira-Silva C, Melo-Rochae G, Pereira ML, Rocha A. Diabetes gestacional: determinação de fatores de risco para diabetes mellitus. Rev Port Endocrinol Diabetes Metab. 2015;10(1):6.

[28] Miailhe G, Kayem G, Girard G, Legardeur H, Mandelbrot L. Selective rather than universal screening for gestational diabetes mellitus? Eur J Obstet Gynecol Reprod Biol. 2015;191:95-100. 10.1016/j.ejogrb.2015.05.003 26112365

[29] Basraon SK, Mele L, Myatt L, Roberts JM, Hauth JC, Leveno KJ, et al. Relationship of Early Pregnancy Waist-to-Hip Ratio versus Body Mass Index with Gestational Diabetes Mellitus and Insulin Resistance. Am J Perinatol. 2016;33(1):114-21. 10.1055/s-0035-1562928 PMC5283057 26352680

[30] Cho GJ, Park JH, Lee H, Yoo S, Shin SA, Oh MJ. Prepregnancy Factors as Determinants of the Development of Diabetes Mellitus After First Pregnancy. J Clin Endocrinol Metab. 2016;101(7): 2923-30. 10.1210/jc.2015-3761 27159192

[31] Shahbazian H, Nouhjah S, Shahbazian N, Jahanfar S, Latifi SM, Aleali A, et al. Gestational diabetes mellitus in an Iranian pregnant population using IADPSG criteria: Incidence, contributing factors and outcomes. Diabetes Metab Syndr. 2016;10(4):242-6. 10.1016/j.dsx.2016.06.019 27350363

[32] Zhang C, Ning Y. Effect of dietary and lifestyle factors on the risk of gestational diabetes: review of epidemiologic evidence. Am J Clin Nutr. 2011;94(6 Suppl):1975S-9S. 10.3945/ajcn.110.001032 PMC3364079 21613563

[33] Ben-Haroush A, Yogev Y, Hod M. Epidemiology of gestational diabetes mellitus and its association with Type 2 diabetes. Diabet Med. 2004;21(2):103-13. 10.1046/j.1464-5491.2003.00985.x 14984444

[34] Ryckman KK, Spracklen CN, Smith CJ, Robinson JG, Saftlas AF. Maternal lipid levels during pregnancy and gestational diabetes: a systematic review and meta-analysis. BJOG. 2015;122(5): 643-51. 10.1111/1471-0528.13261 25612005

[35] Dunlop W. Serial changes in renal haemodynamics during normal human pregnancy. Br J Obstet Gynaecol. 1981;88(1):1-9. 10.1111/j.1471-0528.1981.tb00929.x 7459285

[36] Cheung KL, Lafayette RA. Renal physiology of pregnancy. Adv Chronic Kidney Dis. 2013;20(3):209-14. 10.1053/j.ackd.2013.01.012 PMC4089195 23928384

[37] Mogensen CE. Microalbuminuria as a predictor of clinical diabetic nephropathy. Kidney Int. 1987;31(2):673-89. 10.1038/ki.1987.50 3550239

[38] Parving HH, Viberti GC, Keen H, Christiansen JS, Lassen NA. Hemodynamic factors in the genesis of diabetic microangiopathy. Metabolism. 1983;32(9):943-9. 10.1016/0026-0495(83)90210-x 6350816

[39] Seghieri G, Breschi MC, Anichini R, De Bellis A, Alviggi L, Maida I, et al. Serum homocysteine levels are increased in women with gestational diabetes mellitus. Metabolism. 2003;52(6):720-3. 10.1016/s0026-0495(03)00032-5 12800097

[40] Yoo TW, Sung KC, Shin HS, Kim BJ, Kim BS, Kang JH, et al. Relationship between serum uric acid concentration and insulin resistance and metabolic syndrome. Circ J. 2005;69(8):928-33. 10.1253/circj.69.928 16041161

[41] Santos IC, Frigeri HR, Rea RR, Almeida AC, Souza EM, Pedrosa FO, et al. The glucokinase gene promoter polymorphism -30G>A (rs1799884) is associated with fasting glucose in healthy pregnant women but not with gestational diabetes. Clin Chim Acta. 2010;411(11-12):892-3. 10.1016/j.cca.2010.03.011 20227404

[42] Frayling TM, Timpson NJ, Weedon MN, Zeggini E, Freathy RM, Lindgren CM, et al. A common variant in the FTO gene is associated with body mass index and predisposes to childhood and adult obesity. Science. 2007;316(5826):889-94. 10.1126/science.1141634 PMC2646098 17434869

[43] Dina C, Meyre D, Gallina S, Durand E, Korner A, Jacobson P, et al. Variation in FTO contributes to childhood obesity and severe adult obesity. Nat Genet. 2007;39(6):724-6. 10.1038/ng2048 17496892

[44] Martins MC, Trujillo J, Farias DR, Struchiner CJ, Kac G. Association of the FTO (rs9939609) and MC4R (rs17782313) gene polymorphisms with maternal body weight during pregnancy. Nutrition. 2016;32(11-12):1223-30. 10.1016/j.nut.2016.04.009 27377581

[45] Wang L, Yu Q, Xiong Y, Liu L, Zhang X, Zhang Z, et al. Variant rs1421085 in the FTO gene contribute childhood obesity in Chinese children aged 3-6 years. Obes Res Clin Pract. 2013;7(1):e14-22. 10.1016/j.orcp.2011.12.007 24331679

[46] Bressler J, Kao WH, Pankow JS, Boerwinkle E. Risk of type 2 diabetes and obesity is differentially associated with variation in FTO in whites and African-Americans in the ARIC study. PloS One. 2010;5(5):e10521. 10.1371/journal.pone.0010521 PMC2873943 20502638

[47] Salopuro T, Pulkkinen L, Lindstrom J, Eriksson JG, Valle TT, Hamalainen H, et al. Genetic variation in leptin receptor gene is associated with type 2 diabetes and body weight: The Finnish Diabetes Prevention Study. Int J Obes (Lond). 2005;29(10): 1245-51. 10.1038/sj.ijo.0803024 15997246

[48] Etemad A, Ramachandran V, Pishva SR, Heidari F, Aziz AF, Yusof AK, et al. Analysis of Gln223Agr polymorphism of Leptin Receptor Gene in type II diabetic mellitus subjects among Malaysians. Int J Mol Sci. 2013;14(9):19230-44. 10.3390/ijms140919230 PMC3794830 24051404

[49] Tabassum R, Mahendran Y, Dwivedi OP, Chauhan G, Ghosh S, Marwaha RK, et al. Common variants of IL6, LEPR, and PBEF1 are associated with obesity in Indian children. Diabetes. 2012;61(3):626-31. 10.2337/db11-1501 PMC3282821 22228719

[50] Meirhaeghe A, Amouyel P. Impact of genetic variation of PPARgamma in humans. Mol Genet Metab. 2004;83(1-2):93-102. 10.1016/j.ymgme.2004.08.014 15464424

[51] Groop L, Pociot F. Genetics of diabetes--are we missing the genes or the disease? Mol Cell Endocrinol. 2014;382(1):726-39. 10.1016/j.mce.2013.04.002 23587769

[52] Tok EC, Ertunc D, Bilgin O, Erdal EM, Kaplanoglu M, Dilek S. PPAR-gamma2 Pro12Ala polymorphism is associated with weight gain in women with gestational diabetes mellitus. Eur J Obstet Gynecol Reprod Biol. 2006;129(1):25-30. 10.1016/j.ejogrb.2006.03.016 16678327

[53] Heude B, Pelloux V, Forhan A, Bedel JF, Lacorte JM, Clement K, et al. Association of the Pro12Ala and C1431T variants of PPARgamma and their haplotypes with susceptibility to gestational diabetes. J Clin Endocrinol Metab. 2011;96(10):E1656-60. 10.1210/jc.2011-0381 21795447

[54] Cho YM, Kim TH, Lim S, Choi SH, Shin HD, Lee HK, et al. Type 2 diabetes-associated genetic variants discovered in the recent genome-wide association studies are related to gestational diabetes mellitus in the Korean population. Diabetologia. 2009;52(2):253-61. 10.1007/s00125-008-1196-4 19002430

[55] Shaat N, Ekelund M, Lernmark A, Ivarsson S, Nilsson A, Perfekt R, et al. Genotypic and phenotypic differences between Arabian and Scandinavian women with gestational diabetes mellitus. Diabetologia. 2004;47(5):878-84. 10.1007/s00125-004-1388-5 15095040

[56] Papadopoulou A, Lynch KF, Shaat N, Hakansson R, Ivarsson SA, Berntorp K, et al. Gestational diabetes mellitus is associated with TCF7L2 gene polymorphisms independent of HLA-DQB1*0602 genotypes and islet cell autoantibodies. Diabet Med. 2011;28(9):1018-27. 10.1111/j.1464-5491.2011.03359.x PMC3170100 21672010

[57] NCBI. dbSNP Short Genetic Variantions 2013 [Available from: http://www.ncbi.nlm.nih.gov/SNP/snp_ref.cgi?searchType=adhoc_search&type=rs&rs=rs5393 .

[58] OMIM. Online Mendelian Inheritance in Man 2014 [Available from: http://www.omim.org/ .

[59] Solak M, Ozdemir Erdogan M, Yildiz SH, Ucok K, Yuksel S, Arikan Terzi ES, et al. Association of obesity with rs1421085 and rs9939609 polymorphisms of FTO gene. Molecular biology reports. 2014;41(11):7381-6. 10.1007/s11033-014-3627-2 25074273

[60] Harbron J, van der Merwe L, Zaahl MG, Kotze MJ, Senekal M. Fat mass and obesity-associated (FTO) gene polymorphisms are associated with physical activity, food intake, eating behaviors, psychological health, and modeled change in body mass index in overweight/obese Caucasian adults. Nutrients. 2014;6(8):3130-52. 10.3390/nu6083130 PMC4145299 25102252

[61] Phillips CM, Goumidi L, Bertrais S, Field MR, Ordovas JM, Cupples LA, et al. Leptin receptor polymorphisms interact with polyunsaturated fatty acids to augment risk of insulin resistance and metabolic syndrome in adults. J Nutr. 2010;140(2):238-44. 10.3945/jn.109.115329 20032477

[62] Gotoda T, Manning BS, Goldstone AP, Imrie H, Evans AL, Strosberg AD, et al. Leptin receptor gene variation and obesity: lack of association in a white British male population. Hum Mol Genet. 1997;6(6):869-76. 10.1093/hmg/6.6.869 9175732

[63] Pena G, Guimaraes AL, Veloso RR, Reis TC, Gomes CS, Neto JF, et al. Leptin Receptor Gene Gln223Arg Polymorphism Is Not Associated with Hypertension: A Preliminary Population-Based Cross-Sectional Study. Cardiol Res Pract. 2014;2014:879037. 10.1155/2014/879037 PMC3950908 24772364

[64] Hansen SK, Nielsen EM, Ek J, Andersen G, Glumer C, Carstensen B, et al. Analysis of separate and combined effects of common variation in KCNJ11 and PPARG on risk of type 2 diabetes. J Clin Endocrinol Metab. 2005;90(6):3629-37. 10.1210/jc.2004-1942 15797964

[65] Catalano M, Cortelazzo A, Santi R, Contino L, Demicheli M, Yilmaz Y, et al. The Pro12Ala polymorphism of peroxisome proliferator-activated receptor-gamma2 gene is associated with plasma levels of soluble RAGE (Receptor for Advanced Glycation Endproducts) and the presence of peripheral arterial disease. Clin Biochem. 2008;41(12):981-5. 10.1016/j.clinbiochem.2008.05.007 18538667

[66] Chon SJ, Kim SY, Cho NR, Min DL, Hwang YJ, Mamura M. Association of variants in PPARgamma(2), IGF2BP2, and KCNQ1 with a susceptibility to gestational diabetes mellitus in a Korean population. Yonsei Med J. 2013;54(2):352-7. 10.3349/ymj.2013.54.2.352 PMC3575978 23364967

[67] Cheng Y, Ma Y, Peng T, Wang J, Lin R, Cheng HD. [Genotype discrepancy between maternal and fetal Pro12Ala polymorphism of PPARG2 gene and its association with gestational diabetes mellitus]. Zhonghua Fu Chan Ke Za Zhi. 2010;45(3):170-3. 20450751

[68] Mayans S, Lackovic K, Lindgren P, Ruikka K, Agren A, Eliasson M, et al. TCF7L2 polymorphisms are associated with type 2 diabetes in northern Sweden. Eur J Hum Genet. 2007;15(3):342-6. 10.1038/sj.ejhg.5201773 17245407

[69] Pagan A, Sabater-Molina M, Olza J, Prieto-Sanchez MT, Blanco-Carnero JE, Parrilla JJ, et al. A gene variant in the transcription factor 7-like 2 (TCF7L2) is associated with an increased risk of gestational diabetes mellitus. Eur J Obstet Gynecol Reprod Biol. 2014;180:77-82. 10.1016/j.ejogrb.2014.06.024 25048152

